# Supplementary material for: Reactive Oxygen Species in the Aorta and Perivascular Adipose Tissue Precedes Endothelial Dysfunction in the Aorta of Mice with a High-Fat High-Sucrose Diet and Additional Factors
Source: Int J Mol Sci. 2023 Mar 30;24(7):6486. doi: 10.3390/ijms24076486 (PMC10095299; doi:10.3390/ijms24076486)
Supplement: Supplementary file 1 [file ijms-24-06486-s001.zip › ijms-2274320-supplementary.pdf]

Supplemental Tables and Figures

Reactive Oxygen Species in the Aorta and Perivascular Adipose Tissue Precedes Endothelial Dysfunction in the Aorta of Mice with a High-Fat High-Sucrose Diet and Additional Factors

Ayumu Osaki, Kazuki Kagami, Yuki Ishinoda, Atsushi Sato, ToyokazuKimura, Shunpei Horii, Kei Ito, Takumi Toya, Yasuo Ido, Takayuki Namba, Nobuyuki Masaki, Yuji Nagatomo *, and Takeshi Adachi *

Department of Internal Medicine I, Division of Cardiovascular Medicine, National Defense Medical College,

[#] Corresponding authors

Supplemental Table S1. Systolic Blood Pressure and Heart Rate

	ND	HFHSD	HFHSD-DM	HFHSD-HS	HFHSD-Chol
Systolic BP (mmHg)	103.6 ± 4.8	123.3 ± 4.0*	123.1 ± 2.2*	122.5 ± 3.1*	131.1 ± 3.7*
Heart rate (beats/min)	686.9 ± 5.3	707.9 ± 8.1	709.1 ± 11.4	723.1 ± 8.6	706.1 ± 15.3

Data are presented as mean±SE with n=12-16 per group. ND indicates normal diet group; HFHSD, high-fat/high-sucrose (HFHSD) diet group; HFHSD-DM, HFHSD group, which were received streptozocin (STZ) (50 mg/kg/day for 2 days on the age of 20 weeks); HFHSD-HS, choline deficient HFHSD group; HFHSD-Chol, 1% cholesterol and 0.1% deoxycholic acid containing HFHSD group; GWAT, gonadal white adipose tissue (WAT); SWAT, subcutaneous white adipose tissue; PVAT, perivascular adipose tissue; BP, blood pressure. *$p < 0.05$ compared to ND.

Supplemental table S2. Composition of Normal Diet (ND) and High-Fat and High-Sucrose Diet (HFHSD)

**Composition of ND**	g/100 g	kcal, %
Total calories	343 kcal	
Protein	17.7 g	20.6
Fat	3.8 g	10.0
Carbohydrate	59.4 g	69.4

**Composition of HFHSD**	g/100 g	kcal, %
Total calories	481 kcal	
Protein	20.7 g	17.2
Fat	29.1 g	54.4
Carbohydrate	34.0 g	28.3

**Ingredient**			
Casein	25.0%	Cellulose	5.0%
α-Corn Starch	14.869%	Vitamin Mix (AIN-93)	1.0%
Sucrose	20.0%	Mineral Mix (AIN-93G)	3.5%
Beef Tallow	14.0%	Choline Bitartrate	0.25%
Lard	14.0%	tert-Butylhidroquinone	0.006%
Soybean Oil	2.0%	L-Cystine	0.375%

Supplemental Table S3. Abbreviation Lists

ACh	acetylcholine
ALT	alanine aminotransferase
CVD	cardiovascular diseases
DHE	dihydroethidium
EDR	endothelium-dependent relaxation
ELISA	enzyme linked immunosorbent assay
eNOS	endothelial nitric oxide synthase
FFA	free fatty acid
GWAT	gonadal fat
HDL-C	high-density lipoprotein cholesterol
HFHSD	high-fat and high-sucrose diet
HFHSD-Chol	HFHSD containing 1% cholesterol and 0.1% deoxycholic acid
HFHSD-DM	HFHSD-fed mice treated with streptozocin to develop diabetes
HFHSD-SH	steatohepatitis choline-deficient HFHSD
IRS	insulin receptor substrate
ITT	Insulin tolerance test
Mets	Metabolic syndrome
NAFLD	non-alcoholic fatty liver disease
ND	normal diet
NO	nitric oxide
OGTT	Oral glucose tolerance test
PI3K	phosphatidylinositol-3-kinase
PVAT	perivascular adipose tissue
ROS	Reactive oxygen species
SD	standard deviation
SEM	standard error of the mean
STZ	streptozocin
SWAT	subcutaneous fat
T2DM	type 2 diabetes mellitus
TG	triglycerides
TNF-α	tumor necrosis factor-alpha

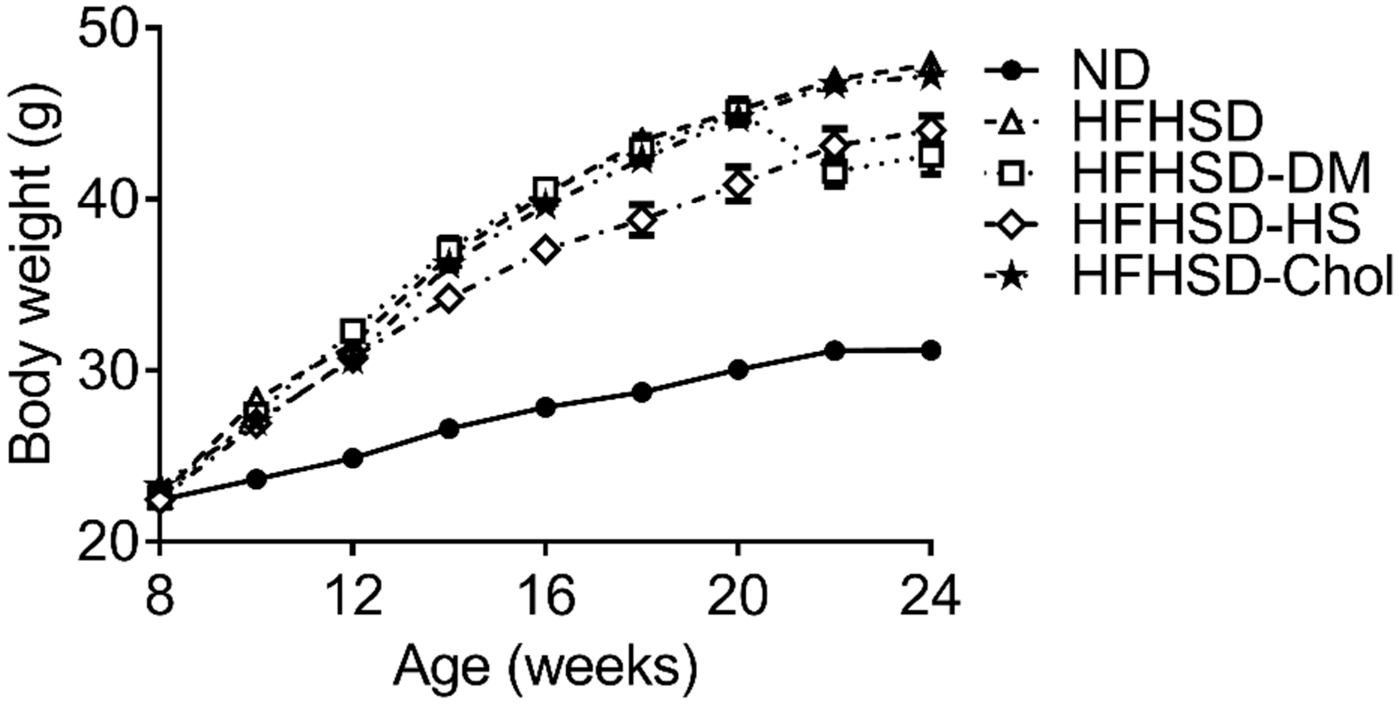

Supplementary Figure S1. Body weight curves for ND, HFHSD, HFHSD-DM, HFHSD-HS, HFHSD-Chol.

The body weight curves are expressed as mean±SEM, respectively (n = 12-16). ND, normal diet group; HFHSD, high-fat/high-sucrose (HFHSD) diet group; HFHSD-DM, HFHSD group, which were received streptozocin (STZ) (50 mg/kg/day for 2 days on the age of 20 weeks); HFHSD-HS, choline deficient HFHSD group; HFHSD-Chol, 1% cholesterol and 0.1% deoxycholic acid containing HFHSD group.
